# Occurrence and preliminarily environmental risk assessment of selected pharmaceuticals in the urban rivers, China

**DOI:** 10.1038/srep34928

**Published:** 2016-10-07

**Authors:** Haidong Zhou, Tianqi Ying, Xuelian Wang, Jianbo Liu

**Affiliations:** 1 School of Environment and Architecture, University of Shanghai for Science and Technology, Shanghai 200093, China

## Abstract

Twelve selected pharmaceuticals including antibiotics, analgesics, antiepileptics and lipid regulators were analysed and detected in water samples collected from 18 sampling sections along the three main urban rivers in Yangpu District of Shanghai, China during four sampling campaigns. Besides, algal growth inhibition test was conducted to preliminarily assess the eco-toxicology induced by the target pharmaceuticals in the rivers. Mean levels for most of target compounds were generally below 100 ng/L at sampling sections, with the exception of caffeine and paracetamol presenting considerably high concentration. The detected pharmaceuticals in the urban rivers ranged from <LOQ for propranolol to 8571 ng/L for caffeine. Qiujiang River could be regarded as the most polluted according to total detected pharmaceutical concentrations. The target pharmaceuticals varied and fluctuated irregularly from the upstreams to the downstreams of the three rivers, indicating the wastewater inputs from non-point sources and their individual different characteristics of transference and transportation. Preliminary eco-toxicological risk assessment showed that the presence of azithromycin, clarithromycin and caffeine may present an ecotoxicological risk in the urban rivers. For other tested pharmaceuticals the inhibition effects of single substances in the urban aquatic environment, based on the algae inhibition tests, were very imperceptible.

Pharmaceuticals have been receiving considerable concerns about their environmental fate and eco-toxicological properties over the last two decades^1^,^2^. Pharmaceutical residuals discharged into the aquatic environment may cause unexpected and harmful consequences on human health and aquatic organisms because they have been specially designed to exert biological effects even at very low concentration levels^3^. Numerous pharmaceuticals and their metabolites have been introduced in urban aquatic ecosystems, mainly originating from effluents of wastewater treatment plants^4^, as these micro-pollutants could not be completely removed by the conventional treatment processes adopted in the plants^5^,^6^,^7^. Besides, pharmaceuticals and their metabolites can directly enter into the urban aquatic environment through urban runoff, scattered untreated sewage discharge, etc. The pharmaceuticals that are frequently present in the urban aquatic environment, originating from effluent of wastewater treatment plants or non-point source sewage has been referred to as wastewater-marking pharmaceuticals^8^. They could reflect the status or trend of wastewater contamination. For example, anti-inflammatory ibuprofen and diclofenac, antibiotics azithromycin and clarithromycin, antipyretic analgesics paracetamol, and psychomotor stimulants caffeine could be regarded as wastewater tracers^8^.

So far, a number of studies have reported the presence of various pharmaceuticals in different aquatic environments^9^,^10^,^11^. Concentrations of pharmaceuticals reported in ground and surface water ranged from a few nanograms per liter up to micrograms per liter^12^,^13^,^14^,^15^. López-Serna *et al*.^12^ presented the occurrence of 72 pharmaceuticals in groundwaters underlying the city of Barcelona, Spain, and antibiotics were the most frequently detected compounds at levels reaching 1000 ng/L. A few drugs also had a high detection concentrations such as ibuprofen (988 ng/L), carbamazepine (136 ng/L), salicylic acid (620 ng/L), azithromycin (1620 ng/L) while some had high detection frenquencies but relatively low concentrations such as clarithromycin (21 ng/L with 100% frenquence) and fenofibrate (29 ng/L with 100% frenquency). In addition to this large amount of analytical data, assessment on the possible ecotoxicological effects of detected pharmaceutical has also been carried out^10^,^16^,^17^,^18^. The risk quotient (RQ) was adopted as a useful tool to characterize potential ecological risk of many contaminants in aquatic ecosystems^19^. Camacho-Muñoz *et al*.^16^ investigated 16 pharmaceuticals in river sediments from Doñana National Park, Spain. The estimated RQ values were higher under chronic toxicity studies than under acute toxicity studies, except in the case of naproxen. The ecotoxicological risk assessment showed that the presence of all studied pharmaceuticals involved a high toxicological risk at short and long term to Doñana ecosystem.

Shanghai is the largest developed and urbanized city in China. With a population of more than 23 millions, pharmaceuticals have been widely used in the hospitals, livestock productions and urban environmental hygiene. Although pharmaceuticals had been detected ubiquitously at concentrated swine farm effluents, aquaculture farm effluents and domestic effluents around rivers, most studies concentrated on Huangpu River, the largest tributary of Yangtze River, which serves as the drinking water source of the city and supplies more than 7.5 million tons of raw drinking water per day^2^,^10^,^13^. As an important part of the aquatic environment, urban rivers have a significant impact on the landscape and the urban ecological environment. They are often responsible for effluent discharge from wastewater treatment plants, urban rainwater collection, flood drainage, and urban landscape and entertainment functions. Seldom studies have been involved in pharmaceuticals present in urban inland rivers which are usually small tributaries of Huangpu River. However, to some extent, small tributaries generally show higher pollutant concentrations than the main river, due to lower dilution of effluents discharged and high density of population^11^.

In this paper 12 representative pharmaceuticals were selected, based on a previous established screening system^8^. The selection took into account academic concerns about them and their concentrations present in urban aquatic environment, as well as their properties of accumulation, persistence, eco-toxicity and related environmental risks caused by them. The selected pharmaceuticals could be also justified partly from the report of Wen *et al*.^2^. The occurrence and variations of the pharmaceuticals in three main urban rivers in Yangpu District of northeast Shanghai, China were investigated. Besides, the eco-toxicological risk of the pharmaceuticals in the urban rivers was preliminarily assessed via algae growth inhibition test. The main aims were to point out the status of wastewater pollution in the urban rivers according to the pharmaceutical presence and potential environmental risk, and expect to take further measures for the pollution control.

## Results and Discussion

### Overall water quality of the three urban rivers

The conventional water quality indicators of the three urban rivers were analyzed, and results are presented in Table 1. The indicators in different sampling sections of the same river showed that the water quality presented little variations along the river. The three rivers also showed similar current status of water quality. According to the National Environmental Quality Standard for Surface Water, China (GB38383-2002), the values of the main conventional water quality indicators were mostly above the standards of the V category of water body (COD ≤ 40 mg/L, NH_3_-N ≤ 2.0 mg/L, TN ≤ 2.0 mg/L, TP ≤ 0.4 mg/L). Therefore, the three rivers were considered as V category or inferior V category of water body during the sampling period. The water quality was very poor, and these rivers could not take the functions of leisure and entertainment for the around people. The urban rivers demonstrated their possibility of pollution by wastewater.

### Pharmaceuticals in the three urban rivers

**Figure 1:**
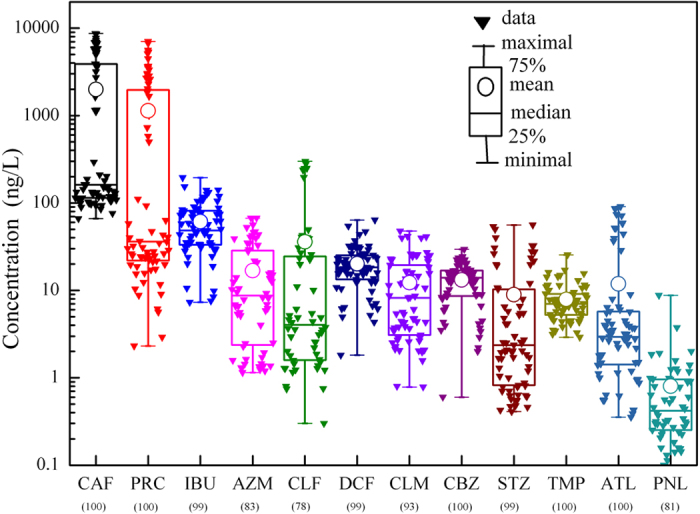
Concentration distribution of the target pharmaceuticals in the three urban rivers. The numbers in the parentheses shows the detection frequency (%) of the target pharmaceuticals.

The target pharmaceuticals in the total 72 composite samples from all sampling sections of the three urban rivers during four sampling campaigns were determined through the aforementioned HPLC-HESI-MS/MS. The concentrations and the frequencies of detection of the pharmaceuticals are summarized in Fig. 1 and Supplementary Table S1. All the 12 target pharmaceuticals were detected, and found ubiquitously in the urban rivers. Five of them including CAF, PRC, CBZ, ATL, and TMP were detected in all the collected samples with a 100% detection frequency while AZM, CLF, and PNL presented a relatively low detection frequency of 83%, 78%, and 81%, respectively. High detection frequencies might be due to their high usage of the pharmaceuticals (such as CAF, PRC, IBU, DCF) or the low degradation rate (such as CBZ).

Average concentrations in the majority of detected samples were generally below 100 ng/L for most target pharmaceuticals, except for several compounds, especially analgesics PRC and psychomotor stimulant CAF, which were present in considerably high concentrations. They were the two predominating pharmaceuticals in all analyzed samples. Their concentrations ranged from 66 ng/L to 8571 ng/L for CAF and 2 ng/L to 7024 ng/L for PRC. On the whole, pharmaceuticals detected in the three urban rivers ranged from <LOQ of PNL to 8571 ng/ L of CAF. For individual pharmaceutical, its detected concentration showed considerably big variation. Besides, the concentrations of most pharmaceuticals were found to be considerably higher during dry weather conditions and significantly decreased during wet weather conditions (Supplementary Table S1), consistent with a significant dilution resulting from rainfall. The time from June to September is of wet-weather period in Shanghai, China. Especially for August, it is the typical rainy and flood month (rainstorm period). From December to next March is the typical dry-weather period. The annually average rainfall in Shanghai is 1172.8 mm, while the rainfall during the wet-weather period accounts for approximately 60%. In other words, the monthly average rainfall is approximately 175.8 mm in this period, 3 times bigger than that in the dry-weather period (58.6 mm). This will result in higher surface runoff and river flow. The higher flow might result in diluting most of pharmaceuticals to lower concentrations than those in the dry-weather period.

The concentrations of the target pharmaceuticals in the urban rivers in this study were almost comparable with or lower than those reported in Europe, North America and other Chinese area. For example, the concentrations of CAF in the surface water were recorded to be maximally 2130 ng/L in Spain^9^ or 7051 ng/L in Beijing, China^20^. The maximal concentrations of CBZ in the present study were lower than those reported in developed countries (566 ng/L in Greece, and 730 ng/L in Germany)^14^,^15^ while the concentrations of PNL were similar to those obtained in Germany (8 ng/L) reported by Kunkel and Radke^15^. Table 2 presents the concentrations of the target pharmaceuticals in the rivers obtained in this study as well as reported in other countries and/or regions.

### Distribution and behavior of pharmaceuticals along the three urban rivers

**Figure 2:**
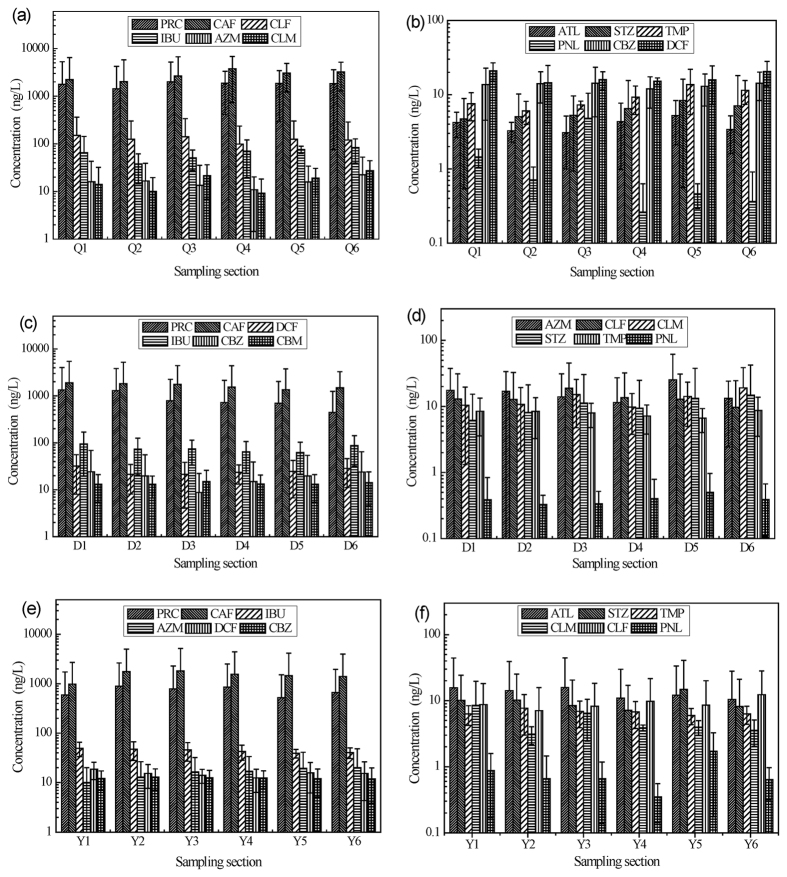
Distribution of the target pharmaceuticals along three urban rivers. (**a,b**) Qiujiang (QJ) River, (**c,d**) Dongzoumatang (DZM) River, and (**e,f**) Yangshupugang (YSP) River of shanghai, China.

The variations of the target pharmaceuticals from the upstream to the downstream along the three urban rivers are shown in Fig. 2, and the data represent statistical values of measurement during four sampling campaigns. All pharmaceuticals could be found at every sampling sections. For QJ River, the concentrations of individual pharmaceutical did not demonstrate the regular variations as is commonly believed that the concentrations are expected to decline from the upstream to the downstream along the river. In fact, most pharmaceuticals showed considerably big fluctuations along the river while some, such as CBZ, varied little. As there is one big hospital nearby between Q3 and Q4 sampling sections, the possible leakage of hospital wastewater and waste drugs might be responsible for the irregular variations of pharmaceuticals^21^. CAF and PRC were the two most abundant pharmaceuticals in the rivers as mentioned above. They were followed by CLF, IBU and DCF. PNL was found lowest in the river. Similar trends of variations of pharmaceuticals were present in the DZM and YSP Rivers. However, IBU, DCF and ATL were the three most abundant compounds, following CAF and PRC. In view of the total concentrations of the studied pharmaceuticals, QJ River contained the highest pharmaceuticals, and could be regarded as the most polluted by wastewater.

The variations of target compound concentrations in the urban rivers could generally be attributed to the biodegradation, sorption, dilution, photodegradation as well as sewage input. The pharmaceuticals were present at relatively fluctuating concentrations or nearly consistent concentrations from the upstream to downstream along the rivers. Also the average flow rates from the upstream to the downstream were roughly estimated, for example, from 16.1 m^3^/s to 18.9 m^3^/s for YSP River, from 8.6 m^3^/s to 10.8 m^3^/s for DZM River, and from 9.1 m^3^/s to 12.8 m^3^/s for QJ River during the normal low water level. Therefore, it appeared that the dilution had little influence on the concentration variations. Pharmaceuticals might undergo biodegradation and adsorption along the rivers^22^. Besides, photodegradation might be effective to eliminate some compounds such as DCF in the rivers^23^,^24^. Moreover, the three urban rivers passed through of the downtown area of Shanghai, receiving pharmaceuticals discharged from some non-point sources. Since 2012, a sewage interception project for the urban rivers has been conducted by Shanghai Municipality. Wastewater discharged from point sources along the rivers has been well collected and prevented to flow into the rivers. However, there were still some outlets of untreated wastewater discharge along the rivers, and even some small-scale garbage heaps spread alongside the rivers, from where the produced leachate might flow into the rivers. However, it would be much more difficult to identify and address those non-point sources discharging to the rivers. According to the estimation of another investigation project on QJ River conducted by our group, the flow of these discharges accounted for about 0.1% of the river flow, however, the pollution contribution from them was more than 50%. The continuous discharge from non-point sources offset the elimination of pharmaceuticals along the river flow, and thus resulted in the irregular variations of pharmaceuticals. As can be seen from QJ River in Fig. 2a, compounds such as CAF, and PRC were present at higher concentrations in the downstream (Q3–Q6) than the upstream (Q1, Q2). As the intervals of the sampling sections were not long due to the length of the urban rivers, and also the non-point source sewage was discharged, the data set of detection may not be easy to describe clearly the temporal variations of pharmaceuticals. Notwithstanding, the selected pharmaceuticals exhibited their long-term presence in the aquatic environment, and therefore could be considered as tracers of wastewater.

### Preliminary ecotoxicological risk assessment of selected pharmaceuticals in the urban rivers

The EC_50_ values of most pharmaceuticals calculated from Sigmoidal (Logistic) model were comparable or similar to those previously reported, and used in the following ecotoxicological risk assessment (Table 3). The values that could not be obtained in the AGIT were referred to the literatures (Table 3). According to the European technical guidance document on risk assessment^25^, compounds can be classified as per their EC_50_-values into different toxic categories: EC_50_ < 1 mg/L, very toxic to aquatic organisms; EC_50_ 1–10 mg/L, toxic to aquatic organisms; and EC_50_ 10–100 mg/L, harmful to aquatic organisms. Compounds with an EC_50_ value above 100 mg/L would not be classified. Most EC_50_ values in the algae test were in the range between 10 and 100 mg/L or even above. Values below 1 mg/L were achieved only for AZM (macrolides antibiotics), and CLM (macrolides antibiotics).

MEC corresponds to the highest concentration of a pharmaceutical measured in the urban rivers during four sampling campaigns, and the RQs of pharmaceuticals are calculated and also presented in Table 3. In general, AZM and CLM could be considered to cause median risk in the urban rivers based on their RQs higher than 0.1. China is now famous not only for a big production but also a large usage of antibiotics. In China, the usage of antibiotics is estimated to be more than 25,000 ton/year^10^. Antibiotics have been extensively used in human therapy, veterinary medicine, and as husbandry growth promoters. AZM and CLM are the two typical macrolides antibiotics extensively used in China, accounting for about 12% and 10% of the usage of antibiotics in Chinese hospitals. For example, according to the pharmaceutical market statistics, the usages of AZM and CLM were estimated to 0.40 t and 0.33 t in 2015 in Hospital 1, one of the biggest hospital in this region. The other two main hospitals listed in Fig. 1 are also the same level as Hospital 1. The three urban rivers pass through populous residential communities, and several small hospitals besides the three listed hospitals are located along the sides of rivers, which might lead to relatively high discharge of the two antibiotics into the rivers. Special attention is suggested to be paid to the ecotoxicological risk of the two antibiotics, and further study on them will be carried on in our group. CAF showed a potential low risk according to its RQ value higher than 0.01. The rest pharmaceuticals showed their negligible environmental risk caused in the aquatic environment. However, the assessment in this study is a preliminary procedure, only taking the effect of individual pharmaceutical into account. In the real aquatic environment, considerable combined effects of pharmaceuticals could occur, and also more ecotoxicity data from chronic studies are needed to assess the environmental risk of pharmaceutical residues and intermediates.

## Conclusions

All target pharmaceuticals were found ubiquitously, and also exhibited their long-term presence in the three urban rivers. Selected pharmaceuticals varied and fluctuated irregularly from the upstreams to the downstreams, suggesting non-point source wastewater inputs along the rivers as well as their individual characteristics of transference and transportation. The pharmaceuticals could be considered as tracers of wastewater.

Caffeine and paracetamol were detected to the highest concentrations with μg/L level in the three urban rivers. The rest were below 100 ng/L. Preliminary ecotoxicological risk assessment showed that azithromycin, clarithromycin and caffeine might cause an ecotoxicological risk in the urban rivers, and for the rest the individual inhibition effects were negligible. Therefore, more concerns about the presence of pharmaceuticals in the urban rivers are proposed to be given, and further studies on them are needed.

## Materials and Methods

### Chemicals and materials

All the reference standards of the selected pharmaceuticals (Supplementary Table S2) were of high-purity grade (>98.5%). The standards azithromycin (AZM), clarithromycin (CLM), sulfathiazole (STZ), trimethoprim (TMP), atenolol (ATL), propranolol (PNL), carbamazepine (CBZ), ibuprofen (IBU), diclofenac (DCF), paracetamol (PRC) and clofibric acid (CLF) were purchased from Dr. Ehrenstorfer (Augsburg, Germany), and the standard of caffeine was supplied by the National Institute of Metrology (China). Internal standards, paracetamol-d_3_ (PRC-d_3_) and sulfathiazole-d_4_ (STZ-d_4_), were obtained from Witega (Germany) and Toronto Research Chemicals (Canada). Standard stock solution of 1000 mg/L for each pharmaceutical was prepared in methanol and stored in the dark at −20 °C. From those, working solutions with mixture of all standard pharmaceuticals were prepared by appropriate dilutions of individual stock solutions in methanol/water (20:80 v/v), and renewed before each analytical run. All the solvents used were HPLC grade or higher. Methanol and acetone were supplied by Merck (Darmstadt, Germany). Milli-Q water was prepared with Aquapro Ultrapure Water System (Beijing, China), and used throughout the study.

Formic acid and ammonium acetate were purchased from Aladdin (Shanghai, China). The two reagents, Na_2_-EDTA and sodium hydroxide (NaOH), chemicals of BG11 Algal medium, and the others used in this study were of analytical grade or better, and purchased from Sigma-Aldrich (USA). Solid-phase extraction (SPE) cartridges (Poly-sery HLB, 200 mg/6 cm^3^) and polyether sulfone syringe filters with 0.22 μm pore size were purchased from ANPEL (Shanghai, China).

### Sampling

**Figure 3:**
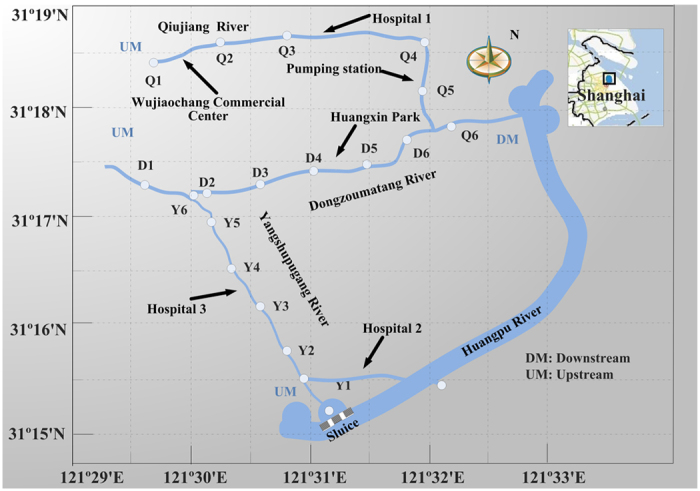
Three main urban rivers and sampling sections in Yangpu District, Shanghai, China. The figure was created by Haidong Zhou using Visio 2007 (Version: Microsoft Office Visio 2007 (12.0.4518.1014) MSo (12.0.4518.1014), https://products.office.com), and the base maps in this figure were generated by Haidong Zhou and Xuelian Wang using the software MapGIS (Version: MapGIS 6.7 build 041010, http://www.mapgis.com.cn (http://english.mapgis.com.cn/en/ for English)).

Samples were collected along the three main urban rivers, i.e. Qiujiang (QJ) River, Dongzoumatang (DZM) River and Yangshupugang (YSP) River, located in Yangpu District of northeast Shanghai City, China (Fig. 3, Supplementary Table S3). Four sampling campaigns were conducted from May 28 to June 2, 2014, September 12, 2014, November 30, 2014 and January 29, 2015, respectively. The sampling dates fell into four seasons, i.e. the summer, the autumn, the winter, and the spring, and could make the campaigns show the occurrence of target pharmaceuticals in all one year. Sampling sections were all set under the bridges across the rivers, considering the convenience and safety. Sampling was conducted about one-third water surface width from the both banks in each sampling section. River water 0.5 m below the water surface was taken. Two samples obtained in each section were mixed to form a composite sample. The composite samples were collected in 4-L amber glass bottles, and transported immediately to the laboratory under cooled conditions (4 °C). Upon reception, samples were filtered through 0.45 μm glass fiber membrane filters and then stored at 4 °C in the dark. Extraction of samples was completed within 24 h.

### Sample preparation

The SPE procedure was performed with Poly-Sery HLB cartridges set on a vacuum 12-position Extraction Manifold from Supelco (USA) to isolation of the compounds from water samples. Each sample volume for extraction was 500 mL. Prior to the extraction, the sample was added with Na_2_EDTA at 0.4 g and the solution pH was adjusted to 7.0 with 1 mol/L NaOH. An amount of 50 ng of internal standard (PRC-d_3_) was also added. Besides, the cartridge was conditioned by 3 × 5 mL of methanol and 3 × 5 mL of Milli-Q water for activation and equilibrium. The sample was then passed through the cartridges with a flow rate of 5 mL/min. After extraction, the cartridge was washed with 4 × 5 ml Milli-Q water in order to remove any interference, and then dried under vacuum for 30 min. Afterwards, it was eluted with 3 × 2 ml of mixture of methanol/dichloromethane/acetone (2:2:1 v/v/v). The eluate was evaporated to dryness under a gentle stream of nitrogen at 40 °C. The dried residue was redissolved by 1 mL of a mixture of methanol/water (20:80 v/v). Finally, the extract was filtered through a 0.22 μm polyether sulfone Syringe filter for further clean-up before analysis.

### Analyses

The target pharmaceuticals were determined by a High-performance Liquid Chromatography System (HPLC, Ultimate3000) from Dionex (USA) combined with a tandem quadrupole Mass Spectrometer equipped with a hot electrospray ionization source (HESI–MS/MS) from Thermo Scientific TSQ Vantage^TM^ (USA).

The HPLC system was used to separate the target pharmaceuticals in the extract. A reversed phase Hypersil GOLD-C18 column (100 mm × 2.1 mm i.d., 1.9 μm particle size) from Thermo (USA) was used for chromatographic separation under a customized mobile phase. The mobile phase was comprised of eluent A (ultra-pure water containing 0.1% formic acid (v/v)) and eluent B (methanol) for HESI in the positive mode (HESI+), and eluent C (ultra-pure water containing 10 mmol/L ammonium acetate) and eluent B (methanol) for HESI in the negative mode (HESI-). A gradient elution program at a flow rate of 0.2 mL/min without any split before entering the source was developed. The elution started with 20% eluent B, was maintained for 1 min, and then increased with a linear gradient from 20% to 50% within 1 min and to 80% over the next 3 min with a curve-2 gradient. Subsequently, the amount of methanol was lowered to 20% in 3 min and maintained for next 5 min. Before the next injection, the system was allowed to equilibrate for 9 min at least. The column temperature was maintained at 30 °C, and the injection volume of the sample extract was 10 μL.

Mass spectrometric analysis was conducted on the HESI-MS/MS system. The source temperature was set at 120 °C. In order to achieve sensitive and selective detection of the analytes, the tandem mass spectrometer (MS/MS) parameters for each analyte—primarily the optimum mode of ionization, the choice of precursor and product ions, and the S-lens RF, and collision energy (Supplementary Table S4)—were optimized using an automatic method development and quantification tool. Each standard compound was optimized to generate a selected reaction monitoring (SRM) experiment, recording the transitions between the precursor ion and the two most abundant product ions. The most abundant transition was used for quantification and the second (if available) for confirmation of the results. Tuning was performed on the ion displaying the weakest signal for each mode of ionization.

Data acquisition was carried out in SRM mode and data processing was done with Thermo Xcalibur software. Detection was done in a HESI+ or HESI- mode. Each target compound was identified using optimal precursor and product ion transitions and comparing the retention time with the corresponding reference standard.

Quantification, based on peak areas of selected ion chromatograms of target compounds, was carried out through internal standard approach. The calibration was conducted from 0.01 to 1000 μg/L (9 points) using the working standard solutions in methanol–water (20:80, v/v) containing all the target pharmaceuticals spiked with 40 ng internal standard STZ-d_4_, and done in triplicate. The correlation coefficients (R^2^) of the calibration curves of all the target compounds were more than 0.99. The method limit of detection (LOD), defined as the concentration that corresponds to three times standard deviation of blanks, was measured by comparison of blank peak areas for each analyte with internal standard STZ-d_4_ in 10 independent replicate analyses. The limit of quantification (LOQ) is the lowest concentration quantified in a sample with acceptable precision under the stated operational conditions of the method. LOQ was determined as 3.3 times of LOD. For the determination of LODs of river water samples, 500 ml ultrapure water was used as the blanks. extracted, and then spiked 40 ng STZ-d_4_ before analysis. Low LOQs were achieved ranging from 0.13 to 3.70 ng/L for the target pharmaceuticals (Table 4). The recoveries of the target compounds were carried out by spiking three replicates of river water samples with standard pharmaceuticals at 50, 200 and 500 ng/L, respectively, with blank subtraction. The recoveries of the pharmaceuticals generally exceeded 75%, and the relative standard deviations (RSDs) were less than 20% (Table 4). Spiked matrices (no less than 50% of the number of samples) were treated and analyzed to determine the recoveries during the measurements to guarantee the precision of quantification. The calculated concentrations of pharmaceuticals were corrected by their recoveries. Besides, procedure blanks and solvent blanks were also treated and analyzed alongside with the measurements.

### Algal growth inhibition test

To assess the eco-toxicological risk of the target pharmaceuticals in the urban rivers, the algae growth inhibition test (AGIT) was introduced following the Guidelines for the Testing of Chemicals—Freshwater Alga and Cyanobacteria Growth Inhibition Test^26^. The test is based on the measurement of growth inhibition of the alga *Selenastrum capricornutum*, renamed as *Pseudokirchneriella subcapitata*. The alga was purchased from FACHB-Collection affiliated with the Institute of Hydrobiology, (Wuhan, China). BG11 Algal medium^27^ was used, and prepared according to the protocol using deionized water and analytical grade chemicals.

The inocula were cultured in two 250-mL Erlenmeyer flasks (100 mL BG11 Algal medium volume) set inside an intelligent illumination incubator (PGX-350B) from BAIDIAN (Shanghai, China). Only cultures in logarithmic phases were used for inoculation. Then, the inocula were added to 250-mL Erlenmeyer flasks (100 mL BG11 Algal medium volume) to obtain an initial algal cell density of 5*10^3^–10^4^ cell/mL. Algal cultures were incubated at 25 ± 1 °C with average ratio of light (4,000 lux) vs. dark at 1. At the beginning of incubation, each pharmaceutical standard with five to seven concentration gradients plus one control (adding the same volume of methanol and water (20:80, v/v) without the compound) was spiked into the cultures to test the algal growth inhibition. The test was conducted in three independent algal culture experiments with quadruplicate samples. For each experiment, cell proliferation was recorded at five time points, i.e. 0, 24 h, 48 h, 72 h, and 96 h during 96 h exposure period. Algal cell density was determined at 650 nm using a 723N UV–visible recording spectrophotometer (Shanghai, China). Measurements were repeated at least three times. The median effective concentration (EC_50_) value of each tested pharmaceutical was calculated from a dose-response curve by a nonlinear curve-fitting procedure—Sigmoidal (Logistic) model.

RQ of a pharmaceutical was calculated as the ratio of its measured environmental concentration (MEC) to its predicted no-effect concentration (PNEC) under the worst case assumptions (Equation (1)).


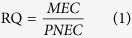


where MEC corresponds to the highest concentration of a pharmaceutical measured in the urban rivers and PNEC is the concentration of a pharmaceutical at which adverse effects are not suspected to occur. In the aquatic environment, PNEC_water_ was calculated using the following Equation 2.


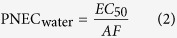


where AF is the safety factor. In this study, EC_50_ values were applied here from the conducted AGIT. A safety factor of 100 was used for long-term/chronic EC_50_ values as this factor value tries to account for the degree of uncertainty in the extrapolation from the test data on a limited number of species compared to the real environment^18^. A frequently used risk ranking criterion proposed by Hernando *et al*.^28^ and de Souza *et al*.^29^ was applied here. When the value of RQ is equal to above 1, an ecological “high risk” for adverse effects caused by the corresponding compound in the aquatic environment is suspected. When the value of RQ is between 0.01 and 0.1, the “low risk” is suspected. The “median risk” is suspected at 0.1 ≤ RQ < 1.

## Additional Information

**How to cite this article**: Zhou, H. *et al*. Occurrence and preliminarily environmental risk assessment of selected pharmaceuticals in the urban rivers, China. *Sci. Rep.* **6**, 34928; doi: 10.1038/srep34928 (2016).

## Supplementary Material

Supplementary Information

## Figures and Tables

**Table 1:** Main conventional water quality indicators of the three urban rivers in Shanghai, China.

River	Sampling site	TOC (mg/L)	COD (mg/L)	TN (mg/L)	NH_3_-N (mg/L)	TP (mg/L)	Turbidity (NTU)	pH
QJ	Q1	17.5 ± 3.5	43.1 ± 20.7	7.3 ± 4.7	3.5 ± 2.9	4.6 ± 1.9	43.8 ± 18.8	7.5–8.1
	Q2	19.1 ± 4.4	47.9 ± 25.2	7.6 ± 4.8	3.7 ± 3.1	4.8 ± 2.0	41.6 ± 19.5	7.4–8.2
	Q3	20.4 ± 3.6	49.7 ± 20.4	9.6 ± 4.6	4.9 ± 2.9	5.5 ± 2.9	40.0 ± 16.1	7.6–8.0
	Q4	22.7 ± 7.4	51.3 ± 5.7	11.7 ± 2.1	6.1 ± 0.7	5.1 ± 4.9	32.2 ± 12.4	7.5–8.2
	Q5	19.0 ± 6.5	49.0 ± 20.8	9.1 ± 3.9	5.0 ± 1.9	5.0 ± 3.0	34.7 ± 17.2	7.3–7.8
	Q6	18.0 ± 4.6	49.4 ± 20.4	9.3 ± 2.4	5.3 ± 0.5	4.4 ± 3.8	39.4 ± 32.2	7.4–8.0
DZM	D1	16.6 ± 1.8	43.5 ± 14.3	8.0 ± 5.4	3.9 ± 3.3	5.2 ± 2.1	44.6 ± 17.9	7.3–7.9
	D2	19.0 ± 3.3	47.4 ± 20.9	7.3 ± 4.7	3.5 ± 3	4.6 ± 1.9	40.2 ± 16.8	7.5–8.0
	D3	18.3 ± 0.9	48.8 ± 10.8	7.2 ± 3.9	4.0 ± 1.9	4.2 ± 2.2	35.1 ± 15.7	7.2–8.1
	D4	16.6 ± 1.3	40.1 ± 10.2	7.8 ± 4.3	3.7 ± 2.7	4.6 ± 2.2	33.6 ± 19.1	7.4–8.1
	D5	15.7 ± 0.4	32.1 ± 22.2	6.9 ± 3.9	3.3 ± 2.4	4.1 ± 2.0	27.5 ± 16.2	7.5–8.2
	D6	18.7 ± 1.4	42.4 ± 16.7	7.8 ± 3.9	4.4 ± 1.8	4.5 ± 2.5	37.4 ± 20.1	7.6–8.3
YSP	Y1	20.3 ± 2.7	52.3 ± 32.8	6.6 ± 3.6	3.1 ± 2.5	4.0 ± 1.8	42.3 ± 16.6	7.5–8.2
	Y2	19.5 ± 4.3	47.5 ± 32.4	7.5 ± 4.7	3.6 ± 3.2	4.8 ± 1.9	45.7 ± 15.9	7.3–8.0
	Y3	20.3 ± 5.1	50.4 ± 34.3	7.2 ± 4.3	3.4 ± 2.9	4.5 ± 1.9	44.1 ± 17.5	7.5–8.3
	Y4	17.4 ± 4.1	34.7 ± 18.5	7.2 ± 4.5	3.6 ± 3.1	4.6 ± 1.8	35.8 ± 18.0	7.4–8.0
	Y5	20.5 ± 2.7	46.4 ± 25.7	6.6 ± 3.8	3.1 ± 2.7	4.1 ± 1.8	37.5 ± 16.1	7.5–7.9
	Y6	20.0 ± 3.0	62.2 ± 27.2	7.0 ± 4.4	3.5 ± 3.0	4.5 ± 1.8	39.8 ± 18.7	7.5–8.2

Note: QJ-Qiujiang River, DZM-Dongzoumatang River, YSP-Yangshupugang River.

**Table 2:** Concentrations of the target pharmaceuticals found in the rivers of this study and other regions/countries reported in the literature.

Compound	Concentration range (ng/L)	Mean concentration (ng/L)	Region/Country	Reference
AZM	n.d.-67	17^a^	This study	
	n.d.-24	4	Rivers adjacent to Jiaozhou Bay, China	18
	n.d.-1620	n.a.	Barcelona, Spain	12
CLM	n.d.-48	12^a^	This study	
	n.d.-85	11	Rivers adjacent to Jiaozhou Bay, China	18
	2-21	n.a.	Barcelona, Spain	12
STZ	n.d.-56	9^a^	This study	
	LOQ-2	n.a.	Alzette and Mess rivers, Luxembourg	30
TMP	3–26	8^a^	This study	
	<5–8	<5	England and Wales	31
	2–12	4	Huangpu river, China	13
	n.d.-9	n.a.	Barcelona, Spain	12
IBU	n.d.-195	61^a^	This study	
	9–2383	n.a.	Alzette and Mess rivers, Luxembourg	30
	LOQ-210	15	Gründlach river, Germany	15
	<LOQ	n.a.	Acheloos River, Greece	14
	<5–36	<5	England and Wales	31
	n.d.-988	n.a.	Barcelona, Spain	12
DCF	n.d.-64	20^a^	This study	
	LOQ-55	n.a.	Alzette and Mess rivers, Luxembourg	30
	<LOD-51	27	Acheloos River, Greece	14
	<10–76	13	England and Wales	31
	n.d.-380	n.a.	Barcelona, Spain	12
CLF	n.d.-299	36^a^	This study	
	105–168	142	Acheloos River, Greece	14
	n.d.-8	n.a.	Barcelona, Spain	12
	<LOD-107	44	Lobregat river basin, Barcelona, Spain	32
PRC	2–7024	1132^a^	This study	
	144–305	259	Acheloos River, Greece	14
	<LOD	n.a.	Barcelona, Spain	12
ATL	1–91	12^a^	This study	
	8–68	18	England and Wales	31
	<LOD	n.a.	Barcelona, Spain	12
PNL	n.d.-9	1^a^	This study	
	1–8	4	Gruündlach river, Germany	15
	n.d.-9	n.a.	Barcelona, Spain	12
CBZ	1–30	13^a^	This study	
	92–186	144	Acheloos River, Greece	14
	<1–25	13	England and Wales	31
	2–128	13	Kaveri River, India	33
	n.d.-136.0	n.a.	Barcelona, Spain	12
CAF	66.3–8570.8	1990^a^	This study	
	37.6–72.2	52	Acheloos River, Greece	14
	54.7–199.0	102	England and Wales	31

Note: n.d.-not detected; n.a.-not available; LOQ-limits of quantification.

^a^Mean concentrations in this study were calculated using the measured values if above the LOQ, the 1/2 LOQ if <LOQ or 0 if not detected.

**Table 3:** EC_50_ and the potential environmental risk assessment of selected pharmaceuticals in the urban rivers.

Compound	EC_50_ (mg/L)	EC_50_ in literatures (mg/L)	PNEC (ng/L)	MEC (ng/L)	RQ	Reference
AZM	0.026	0.015 (susceptible pathogenic bacteria test)	260	67.3	0.26	34
CLM	0.015	0.011 (*P. subcapitata* 96-h growth inhibition)	150	47.7	0.32	35
STZ	90.7	85.4 (*Daphnia magna* 96-h immobilization)	907000	56.0	<0.01	36
TMP	145.5	80.3 (*Selenastrum capricornutum* 72-h test)	1455000	25.5	<0.01	37
IBU	—	342.2 (algae *D. subspicatus* growth inhibition)	3422000	194.6	<0.01	17
DCF	257.1	16.3 (*Pseudokirchneriella subcapitata* growth inhibition)	2571000	55.0	<0.01	38
PRC	—	134.0 (*Scenedesmus subspicatus* 72-h test)	1340000	7023.7	<0.01	1
ATL	45.1	62.0 (algae *D. subspicatus* growth inhibition)	451000	91.0	<0.01	39
PNL	1.7	0.7 (*Desmodesmus subspicatus* 48-h test)	17000	8.7	<0.01	39
CLF	—	89.0 (*Pseudokirchneriella subcapitata* 96-h test)	890000	299.3	<0.01	1
CBZ	133.0	>100 (*Pseudokirchneriella subcapitata* 96-h test)	1330000	29.5	<0.01	3
CAF	53.1	87.5 (fish *Leuciscusidus* 96-h test)	531000	8570.8	0.02	30

**Table 4:** Limits of detection (LOD) and limit of quantification (LOQ), and recoveries (n > 20) of target pharmaceuticals analyzed by HPLC-HESI-MS/MS.

Analyte	LOD (ng/L)	LOQ (ng /L)	50 ng spiked matrices		200 ng spiked matrices		500 ng spiked matrices	
			Recovery (%)	RSD (%)	Recovery (%)	RSD (%)	Recovery (%)	RSD (%)
ATL	0.34	1.12	105.5 ± 8.0	7.6	89.5 ± 9.2	10.3	89.5 ± 11.4	12.7
PRC	0.26	0.86	74.8 ± 2.4	3.2	79.7 ± 3.2	4.0	88.1 ± 4.6	5.2
STZ	0.04	0.13	81.5 ± 2.2	2.7	90.2 ± 11.0	12.2	93.0 ± 9.4	10.1
TMP	0.04	0.13	112.1 ± 4.9	4.4	108.9 ± 2.2	2.0	86.8 ± 11.5	13.2
CAF	0.20	0.66	100.7 ± 8.8	8.7	116.1 ± 2.1	1.8	111.0 ± 10.6	9.5
AZM	0.34	1.12	88.3 ± 3.6	4.1	87.9 ± 3.2	3.6	98.0 ± 4.0	4.1
PNL	0.10	0.33	115.8 ± 2.5	1.9	110.5 ± 1.9	1.7	86.4 ± 9.2	10.6
CBZ	0.10	0.33	91.5 ± 3.3	3.6	84.0 ± 1.0	1.1	80.9 ± 10.7	13.2
CLM	0.08	0.26	113.8 ± 3.9	3.5	84.1 ± 5.3	6.3	118.7 ± 11.8	9.9
CLF	1.08	3.56	86.5 ± 1.6	1.8	82.6 ± 1.8	2.2	85.1 ± 4.0	4.6
DCF	0.14	0.16	87.1 ± 3.4	3.9	82.6 ± 2.9	3.5	95.6 ± 9.7	10.1
IBU	1.12	3.70	85.4 ± 4.2	4.9	87.1 ± 7.8	9.0	90.1 ± 8.9	9.9
