# Supplementary material for: Occurrence and preliminarily environmental risk assessment of selected pharmaceuticals in the urban rivers, China
Source: Sci Rep. 2016 Oct 7;6:34928. doi: 10.1038/srep34928 (PMC5054680; doi:10.1038/srep34928)
Supplement: Supplementary Information [file srep34928-s1.pdf]

SUPPLEMENTARY INFORMATION

Occurrence and preliminarily environmental risk assessment of selected pharmaceuticals in the urban rivers, China

Haidong Zhou*, Tianqi Ying, Xuelian Wang & Jianbo Liu

School of Environment and Architecture, University of Shanghai for Science and Technology, Shanghai 200093, China

* Corresponding author, School of Environment and Architecture, University of Shanghai for Science and Technology, No. 516, Jungong Road, Shanghai 200093, China. Tel/Fax: +86 21 55271722. Email: zhouhaidong@usst.edu.cn.

Table S1. Range, and detection frequency (%) of pharmaceuticals in the three urban rivers during four sampling campaigns.

Sampling campaign	Measurement	ATL	PRC	STZ	TMP	CAF	AZM	PNL	CBM	CLM	CLF	DCF	IBU
1 [a]	Range (ng/L)	0.6-91.1	12.4-728	0.4-8.2	3.8-25.6	85.4-2729.3	16.6-67.3	0.1-3.8	13.9-29.5	2.6-41.5	19.9-299.3	12.3-43.5	31-116.9
	FD [e] (%)	100	100	100	100	100	100	100	100	100	100	100	100
2 [b]	Range (ng/L)	2.4-7.9	2.3-1905.8	2-7.9	5.7-14.9	114.5-6868.8	n.d.[f]-8.3	n.d.-1.2	12.5-17.8	2-47.7	n.d.-11	15.1-54.4	n.d.-140.2
	FD (%)	100	100	100	100	100	94	72	100	100	61	100	94
3 [c]	Range (ng/L)	0.4-9.0	19.6-2744.7	n.d.-1.9	2.9-12.9	66.3-3888.0	n.d.-15.4	n.d.-8.8	8.6-16.1	0.8-39.8	1.2-5.1	5-32.1	7.3-58.6
	FD (%)	100	100	94	100	100	83	83	100	100	100	100	100
4 [d]	Range (ng/L)	0.9-3.7	1658.8-7023.7	10.3-56	5.3-16	3569.7-8662.4	n.d.-38.9	n.d.-2	0.6-8.9	n.d.-41	n.d.-3.8	1.8-63.8	43.4-194.6
	FD (%)	100	100	100	100	100	56	67	100	72	56	100	100

Note: [a] From May 28 to June 2, 2014, [b] September 12, 2014, [c] November 30, 2014, [d] January 29, 2015, [e] Frequency of detection (%), [f] Not detected (<LOQ).

Table S2. Physicochemical properties of the target pharmaceuticals in this study.

Compounds	Therapeutic groups	CAS No.	Molecular formula	Molecular Weight	Molecule structure	logKow	pKa
Azithromycin (AZM)	macrolides antibiotics	83905-01-5	$C_{38}H_{72}N_2O_{12}$	749.0		4.02 [a]	8.7 [a]
Clarithromycin (CLM)	macrolides antibiotics	81103-11-9	$C_{38}H_{69}NO_{13}$	748.0		3.16 [a]	8.99 [e]
Sulfathiazole (STZ)	Antimicrobial sulfonamides	72-14-0	$C_9H_9N_3O_2S_2$	255.3		0.89 [d]	7.29 [d]
Trimethoprim (TMP)	Broad spectrum antibiotics	738-70-5	$C_{14}H_{18}N_4O_3$	290.3		0.91 [d]	7.12 [d]
Ibuprofen (IBU)	Anti-inflammatory/ analgesic drugs	15687-27-1	$C_{13}H_{18}O_2$	206.3		3.97 [a]	4.91 [a]
Diclofenac (DCF)	Non steroidal anti-inflammatory drugs (NSAID)	15307-79-6(Na) 15307-86-5	$C_{10}H_{11}ClO_3$	214.6		4.51 [a]	4.15 [a]

Paracetamol (PRC)	Antipyretic analgesics	103-90-2	$C_8H_9NO_2$	151.2	0.46 [a]	9.38 [a]
Atenolol (ATL)	Cardioselective beta blockers	29122-68-7	$C_{14}H_{22}N_2O_3$	266.3	0.16 [a]	9.6 [e]
Propranolol (PNL)	Beta-blockers	525-66-6	$C_{16}H_{21}NO_2$	259.4	3.48 [a]	9.1 [b]
Clofibric acid (CLF)	Lipid regulators	882-09-7	$C_{10}H_{11}ClO_3$	214.6	2.57 [b]	3.2 [b]
Carbamazepine (CBZ)	Psychiatrics-antiepileptics	298-46-4	$C_{15}H_{12}N_2O$	236.3	2.45 [d]	7.0 [d]
Caffeine (CAF)	Psychomotor stimulants	58-08-2	$C_8H_{10}N_4O_2$	194.2	−0.07 [a]	10.4 [a]

Note: [a] Data from Kosma et al.[1]; [b] Data from Camacho- Muñoz et al.[2]; [c] Data from Zhang et al.[3]; [d] Data from Kim et al.[4]; [e] Data from the manufacturers.

Table S3. Details of sampling along Qiujiang (QJ) River, Dongzoumatang (DZM) River and Yangshupugang (YSP) River in Shanghai, China.

River	Flow rate (m^3/s)	Length (km)	Width (m)	Depth (m)	Sampling section	Sampling campaign
**QJ**	5.4-24.0	7.0	30-40	0.8-1.8 [a]<br>3.9-4.0 [b]	Q1-Q6	May 28 to June 2, 2014<br>September 12, 2014<br>November 30, 2014<br>January 29, 2015
**DZM**	4.8-20.5	5.0	20-30	2.2-2.4 [a]<br>3.8-4.0 [b]	D1-D6	
**YSP**	14.2-28.3	4.5	~27	~3.5	Y1-Y6	

Note: [a] Depth during the normal low water level; [b] Depth during the normal high water level.

Table S4. Retention times and their optimal MS/MS parameters using hot electrospray ionization of target pharmaceuticals.

Compound	Mw (Da)	Precursor ion (m/z)	Product ion (m/z)	Collision energy (eV)	S-Lens RF (v)	Ionization mode	Retention time (min)
**PNL**	259.4	260.2	116.1 *	17	85	HESI+	5.93
			183.1	17	85		
**AZM**	749.0	749.5	591.5 *	24	163	HESI+	5.32
			158.1	37	79		
**CBZ**	236.3	237.1	194.1 *	19	77	HESI+	6.61
			193.1	33	77		
**CLM**	748.0	748.4	158.1 *	26	129	HESI+	7.14
			590.5	14	129		
**STZ**	255.3	256.0	156.0 *	14	71	HESI+	2.72
			108.1	23	71		
**TMP**	290.3	291.1	230.1 *	23	109	HESI+	3.74
			261.1	24	109		
**ATL**	266.3	267.1	145.1 *	25	86	HESI+	1.80
			190.1	17	86		
**PRC**	151.2	152.0	110.1 *	15	66	HESI+	2.44
			65	29	66		
**CAF**	194.2	195.1	138.1 *	19	79	HESI+	3.98
			110.1	33	79		
**IBU**	206.3	205.0	161.0 *	7	42	HESI-	7.32
**CLF**	214.6	213.0	127.0 *	20	53	HESI-	5.98
**DCF**	296.2	294.0	250.1 *	14	59	HESI-	7.15
			214.1	21	59		
**PRC-d_3**	154.2	155.1	111.1*	15	74	HESI+	2.43
			93.1	21	74		
		153.1	107.1*	19	57	HESI-	2.38
			118.1	34	57		
**STZ-d_4**	259.3	260.0	160.0*	14	84	HESI+	2.68
			96.1	25	84		
		258.0	160.0*	19	58	HESI-	2.56
			98.0	20	58		

Note: ∗ product ion for quantification.

References

1. Kosma, C. I., Lambropoulou, D. A. & Albanis, T. A. Occurrence and removal of PPCPs in municipal and hospital wastewaters in Greece. *J. Hazard. Mater.* **179**(1–3), 804-817 (2010).
2. Camacho-Muñoz, D., Martín, J., Santos, J. L., Aparicio, I. & Alonso, E. Distribution and Risk Assessment of Pharmaceutical Compounds in River Sediments from Doñana Park (Spain). *Water, Air, & Soil Pollution* **224**(10), 1665 (2013).
3. Zhang, N., Liu, G., Liu, H., Wang, Y., He, Z. & Wang, G. Diclofenac photodegradation under simulated sunlight: Effect of different forms of nitrogen and kinetics. *J. Hazard. Mater.* **192**(1), 411-418 (2011).
4. Kim, Y., Choi, K., Jung, J., Park, S., Kim, P.-G. & Park, J. Aquatic toxicity of acetaminophen, carbamazepine, cimetidine, diltiazem and six major sulfonamides, and their potential ecological risks in Korea. *Environ. Int.* **33**(3), 370-375 (2007).
